# A Framework for Estimating the Burden of Chronic Diseases: Design and Application in the Context of Multiple Sclerosis

**DOI:** 10.3389/fneur.2019.00953

**Published:** 2019-09-04

**Authors:** Marco Kaufmann, Milo Alan Puhan, Jens Kuhle, Özgür Yaldizli, Tomas Magnusson, Christian P. Kamm, Pasquale Calabrese, Viktor von Wyl

**Affiliations:** ^1^Department of Epidemiology, Epidemiology, Biostatistics and Prevention Institute, University of Zurich, Zurich, Switzerland; ^2^Neurologic Clinic and Policlinic, Departments of Medicine, Biomedicine and Clinical Research, University Hospital and University of Basel, Basel, Switzerland; ^3^Business Intelligence, MediService, Zuchwil, Switzerland; ^4^Neurology and Neurorehabilitation Centre, Luzerner Kantonsspital, Lucerne, Switzerland; ^5^Department of Neurology, Inselspital, University Hospital Bern and University of Bern, Bern, Switzerland; ^6^Division of Molecular and Cognitive Neuroscience, University of Basel, Basel, Switzerland

**Keywords:** prevalence, benchmark-multiplier, generalizability, external validity, SMSR, epidemiology

## Abstract

**Background:** When population-based databases are unavailable, nationwide assessments of the disease burden of multiple sclerosis (MS) resort to clinical, administrative or convenience-sampled data sources, which may produce results of limited external validity. Our aim was to develop a framework for estimating measures of occurrence of chronic diseases, and more broadly disease burden, that mitigate these limitations and to apply this framework to estimate the prevalence of multiple sclerosis (MS) in Switzerland.

**Methods:** We developed a 7-step framework which implements the combination of several data sources together with a resampling and critical appraisal approach. The framework was applied to estimate the MS prevalence for 2016 in Switzerland, for which four distinct data sources (Swiss MS registry, Swiss national MS treatment registry, MediService database, and Swiss MS cohort study) were combined. Results were reviewed by disease experts and compared to earlier Swiss estimates and current prevalence estimates from other countries.

**Results:** We estimate that in the year 2016 between 14,650 and 15,700 persons with MS have been living in Switzerland, yielding a period prevalence of 174–187/100,000 inhabitants. Compared to the last estimate in 1986, we detected a substantial increase of MS diagnoses which coincides with a higher number of diagnoses in women below the age of 65.

**Conclusions:** Internationally, Switzerland is a high-prevalence country for MS, although estimates were somewhat lower than recent evaluations of Northern European countries. In addition, we corroborate previous reports that the prevalence increase coincides with a higher number of MS diagnoses among women. The proposed framework has wide applicability and the potential to place estimates of disease occurrence and burden with imperfect data availability on more solid grounds.

## Introduction

In the field of Multiple Sclerosis (MS), studies on the prevalence of MS have a long history, and follow a multitude of approaches. In Northern-European countries, for example Denmark, the existence of national health care and disease databases with very high population coverage allow a direct estimation from these data (1). By contrast, in settings without mandatory reporting of new MS diagnoses, some studies have relied on regional, clinic-based estimates (e.g., based on new and existing diagnoses), followed by nation-wide extrapolation (2, 3). Following the second approach, the point prevalence of MS in Switzerland was last thoroughly estimated by Beer and Kesselring to be around 100/100,000 inhabitants (4).

In Switzerland and many other countries, accurate estimation of measures of disease occurrence and burden is a challenging task for several reasons. First, although not considered a rare disease, MS is still not frequent enough to be measured efficiently through random sampling of the general population. This is further complicated by the universal challenges of a sound random sampling (5, 6). Second, changing diagnostic criteria, reclassification of syndromes such as Neuromyelitis Optica Spectrum Disorders (NMOSD) as not being MS and the availability of treatment options altering the disease course [disease-modifying treatments (DMT)] have impacted the definition of MS and hence also epidemiological measures (7–10). Therefore, the comparison of earlier estimations with newer assessments needs to take these changes in MS case definition into account. Third, epidemiological assessments relying on clinical or convenience samples are not always externally valid and could therefore lead to incorrect extrapolations. Against this background, a detailed understanding of inclusion- and selection mechanisms of the analytic databases, as well as of the factors “driving” an outcome of interest is crucial (6, 11, 12).

Despite the emerging literature on statistical methods for assessing and improving external validity, there is very limited guidance for more practically oriented research to assess the representativeness of burden of disease parameters derived from non-randomly sampled (clinical or administrative) databases. Moreover, there has—to our knowledge—never been an attempt for a more unified approach to estimate burden of disease parameters for MS from non- population-based databases. The present study therefore has two aims. First, it aims to provide an update of the prevalence of MS in Switzerland. Second, it will do so by developing a framework approach for comparing and combining data from different datasets and for assessing the external validity.

## Methods

### Available Datasets

The Swiss Multiple Sclerosis Registry (SMSR) is a patient-centered registry that prospectively enrolls and follows adult persons with MS (PwMS) in Switzerland (*n* = 1,911 as of Feb 11, 2019). The Swiss MS registry is an observational study based on self-reported data which is supplemented with detailed clinical data for a subpopulation of ~15%. The registry has specifically been structured to also include PwMS that are usually less likely to participate in clinical studies, for example, people with a new diagnosis, severe impairments or living in areas far from MS centers (13, 14). The Swiss MS registry covers the full spectrum of the Swiss MS population with potential slight under representations in very young and older age groups of PwMS. The Swiss MS registry study was approved by the ethics committee of the canton of Zurich (PB-2016-00894) and written informed consent was obtained from all participants (13). All data were pseudonymised prior to analysis.

This study also had access to the Swiss national MS treatment registry, administered by the Swiss association for joint tasks of health insurances (SVK), which is an administrative database in Switzerland and is used for the reimbursement process of disease-modifying therapies (DMT) for MS for certain insurance companies (15). Due to the mandatory health insurance system in Switzerland with very comprehensive coverage of essential treatments, DMTs are reimbursed fully if drugs are utilized according to formal approval criteria. Therefore, the access to DMT and neurological care in general is unrestricted (15, 16). In consequence, the Swiss national MS treatment registry delivers a good estimate of DMT users in the insurances covered by the Swiss association for joint tasks of health insurances. These insurances had a market share of 67% in 2016, the index year of the study. The database contains anonymized, individual-level DMT reimbursement information which consist for example of the disease course, type of drug or date of diagnosis, filled in by neurologists. The ethics committee of North-West and Central Switzerland (EKNZ UBE Req-2019-00470) has provided a confirmation that the SVK data do not fall under the Swiss Human Research law. The Clinical Trial Unit at the University Hospital of Basel is responsible for the data (17).

MediService is a company in Switzerland that offers a service to handle the entire DMT process, from the prescription to the delivery of DMT, including the payment with the insurance as well as, if applicable, the reimbursement approval by the Swiss association for joint tasks of health insurances (therefore allowing to compare patients who are included in the Swiss national MS treatment registry databases and those who are not). For this study, we used aggregated data of the year 2016.

For validation purposes, we further reviewed data from the Swiss Multiple Sclerosis Cohort study (SMSC). The Swiss MS cohort study is a prospective, clinical cohort of PwMS in Switzerland, which are included at 7 Swiss university hospital settings, and are regularly surveyed. The data collection includes information about disease characteristics, MRI data and body fluid sampling. This study is prospectively following 1,200 PwMS, with a median follow-up duration of 3 years at the end of 2016 (18). For this study, aggregated data until the end of 2016 was used.

### Framework for Estimating the Burden of Chronic Diseases

**Figure 1:**
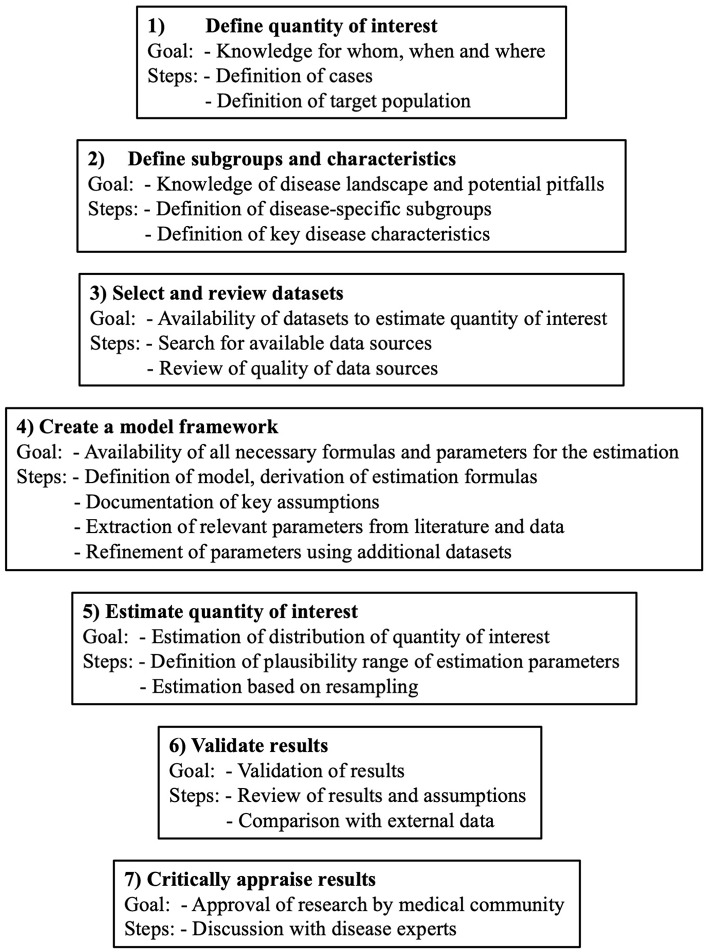
General outline of the 7 steps of the framework to estimate the burden of chronic diseases.

The general outline of the estimation framework is shown in Figure 1. It consists of 7 steps. Limitations in the individual steps, for example non-applicability or limited credibility, do not prohibit the use of the framework. However, the limitations should be considered when defining the estimation uncertainty and clearly highlighted in the discussion. For the present use case, the estimation of MS prevalence in Switzerland, the core estimation process is based on the *benchmark-multiplier* method discussed by Bollaerts et al. (19). For the seven steps presented below, we will, in each step, first introduce the goal and generic idea and then describe its specific implementation for our use case.

#### Step 1 – Define Quantity of Interest

The quantity of interest, often the prevalence, needs to be defined precisely. This definition consists of, among other things, the case definition, the exact definition, and the size of the target population (i.e., to which population the estimate should apply) and the index time.

##### Prevalence of MS

The quantity of interest in our study was the period prevalence of persons with clinically-definite MS in Switzerland in the index year 2016. The target population was the Swiss population in 2016 which consisted of ~8,420,000 (20).

#### Step 2 – Define Subgroups and Characteristics

Step 2 consists of the definition of specific disease population subgroup(s) and key disease characteristics. Specific in this context means that the members of a subgroup can be identified with no or very little misclassification and that the subgroup is reasonably large (>10% of population). Furthermore, the subgroup(s) should be chosen in a way that reliable information on the subgroup size are available. In case more than one subgroup is of interest, the subgroups have to be mutually exclusive. Key disease characteristics are those that play a major role in the characterization of the disease. They can most easily be found by reviewing study population descriptions of previous disease-specific publications.

##### Prevalence of MS

In our use-case we defined our subgroup to be the PwMS taking a DMT in the last 12 months, because we were already aware of the existence of a dataset covering this group. Key disease characteristics were the disease subtype and duration, the treatment status and type, the disability and demographic information.

#### Step 3 – Select and Review Datasets

Step 3 includes the search for and review of available disease-specific datasets and their assessment with regard to suitability and quality. The minimal number of datasets depends on the quantity of interest as well as the chosen estimation method, but in general more datasets are preferable. For example, for the *benchmark-multiplier* method, at least two datasets are needed, one containing absolute group-sizes (*benchmark*), and another one that is population-based and reflects the target population (*multiplier*) (19). The quality of the datasets should be assessed individually by looking at the specific population characteristics, patient selection effects, and potential issues with external validity. Furthermore, the inclusion of the predefined subgroups from step 2 datasets should be verified in the different datasets and compared with respect to key disease characteristics.

##### Prevalence of MS

The following search strategy was applied to our use case. First, we screened the literature for MS studies in Switzerland, as well as the references leading to other studies. Moreover, disease-experts were contacted to learn about existing data sources. This process led to the identification of four main data sources for our study. For the *benchmark* dataset with the absolute group-sizes, the Swiss national MS treatment registry dataset was identified as a suitable database. In addition, the Swiss MS registry was selected as the most suitable dataset, reflecting the target population, the *multiplier* (19). In addition to these, the MediService and the Swiss MS cohort study data were reviewed and obtained for later use. We reviewed the datasets and compared the coinciding subgroups regarding key disease characteristics and found generally good correspondence (cf. results section).

#### Step 4 – Create a Model Framework

This step involves the model specification and the determination of the estimation formulas which highly depend on the quantity of interest. Any assumptions regarding the data, the definitions, and the estimation process should be clearly outlined and assessed in the specified datasets. Furthermore, the existence of all predefined parameters for the estimation needs to be assessed (that is, parameters with assumed or literature-derived values owing to a lack of data). Moreover, by use of additional datasets from step 3, the main databases for estimation should be reviewed regarding possible distributional differences of key disease characteristics. This step is intended to increase the credibility of the data sources and to mitigate observed distributional differences in later analysis steps. In the case of large disagreements between the different data sources, the uncertainty should be reasonably considered in the estimation in step 5. The findings and possible limitations derived from this step should be adequately reflected and addressed in the Discussion section of research reports.

##### Prevalence of MS

The basic approach was the *benchmark-multiplier* method. This method operates by dividing the group size of the specified subgroup (${\hat{N}}_{x}$), so the *benchmark*, by its percentage of the entire population ($\hat{\pi }$), the *multiplier*, as displayed in Formula (1),

(1) $$\begin{array}{r} Prevalence=\;\frac{{\hat{N}}_{x}}{\hat{\pi }}. \end{array}$$

However, as discussed by Bollaerts et al. (19) this estimator is biased and therefore we used the suggested bias-corrected estimator,

(2) $$\begin{array}{r} \;Prevalence=\frac{{\hat{N}}_{x}}{\hat{\pi }}-\frac{1}{n}*{\hat{N}}_{x}*\left(\frac{1}{\hat{\pi }}-1\right), \end{array}$$

with *n* being the sample size of the *multiplier* (19). In particular, a subtraction term is included in the equation, which depends on the sample size of the *benchmark* (${\hat{N}}_{x}$), inversely on the sample size of the *multiplier* dataset ($\frac{1}{n}$) and on the population percentage that is covered by the *multiplier* ($\frac{1}{\hat{\pi }}-\;1$).

The prevalence per 100,000 persons at risk was estimated as follows:

(3) $$\begin{array}{r} Prevalence\;per\;100^{\prime }000=\;\frac{Prevalence}{Population\;at\;risk}*100^{{}^{\prime }}000. \end{array}$$

The key assumptions of the *benchmark-multiplier* method consist of the *benchmark* covering the subgroup exhaustively, the *multiplier* being representative of the target population and the subgroup definition of the *benchmark* exactly coinciding with the corresponding subgroup of the *multiplier* (19).

The parameters for the estimation with the benchmark-multiplier method were available from an augmented Swiss national MS treatment registry dataset and the Swiss MS registry database. The augmented Swiss national MS treatment registry dataset relies on the Swiss national MS treatment registry data, which was complemented by additional (pseudo-) records so as to reflect also the population of PwMS receiving DMT, whose reimbursement applications are not processed by the Swiss association for joint tasks of health insurances. These pseudo-records were constructed on the basis of the MediService database, which includes all types of DMT prescriptions (i.e., those submitted to the Swiss association for joint tasks of health insurances and others that are directly submitted to individual insurers). The exact details of this step, which balanced misrepresentations in the Swiss national MS treatment registry, are shown in the Supplementary Material.

In addition, the Swiss MS registry database was compared to aggregated data from the Swiss MS cohort study (which are not directly used in the benchmark-multiplier estimation) in order to ascertain approximate representativeness of the Swiss MS population and the DMT usage patterns.

#### Step 5 – Estimate Quantity of Interest

In Step 5, the calculations on the basis of Step 4 are executed. For all random parameters, a probability distribution of plausible values reflecting the estimation uncertainty needs to be defined. The distributions and the uncertainty are determined based on the observed variability of key parameters across the different study databases, as well as based on information from the literature. For the calculation process, values for random parameters are repeatedly drawn from the pre-defined distributions and the estimation process is repeated a large number of times. Main outcome results are recorded. The final estimation result is a distribution of main outcome parameters, which reflects the pre-defined assumptions (also regarding literature-based parameters), as well as the uncertainty stemming from distributional differences across the different databases used in the analyses.

##### Prevalence of MS

The review process in steps 3 and 4 indicated uncertainties in two main parameters, which were addressed in the modeling study by defining conservative probability distributions to draw values from. These parameters were (1) the group size of the persons using DMT in 2016 (*benchmark*) and (2) the share of the entire PwMS population this group reflects (i.e., the *multiplier*). Subsequently, the parameters were then drawn from the distributions, and the estimation was executed according to Formulas (2) and (3). This process was repeated 100,000 times. Estimations of the absolute number of PwMS were performed separately for 5-years strata of disease duration, which were then collapsed into a single parameter (overall number of PwMS).

#### Step 6—Validate Results

In step 6, the estimated distributions as well as the modeling assumptions need to be subject to critical review. If available, the results should be validated using other databases that were not included in the estimation process and should ideally stem from the same country and the same period. If unavailable, other databases may be used, but comparability of key assumptions and subgroups must be ascertained.

##### Prevalence of MS

We carefully reviewed the assumptions of our estimation and the obtained distribution of the number of PwMS living in Switzerland. Furthermore, we compared the age- and sex-specific MS prevalence per 100,000 to the values of the estimation of Beer and Kesselring in 1986 (4). Lastly, the MS prevalence per 100,000 of the UK, Sweden, Norway, Denmark, France and Germany were set in context to our results (1, 21–26).

#### Step 7—Critically Appraise Results

The results are critically discussed with disease experts in the last step. Whenever possible, these persons should not be directly involved in the estimation to take a fresh view on the results. In the case of disagreements, the assumptions as well as the datasets should be carefully reviewed. Major concerns by the expert should either be reflected in an improved estimation process and/or highlighted as study limitations in the Discussion section of the research report.

##### Prevalence of MS

As a control step, we defined subgroups based on disease course and DMT usage pattern and determined their group sizes based on occurrence frequencies in the Swiss MS registry. The period prevalence distribution as well as the groups were then critically discussed by the scientific committee of the Swiss MS registry which consists of Swiss MS specialists. They were not directly involved in the estimation.

### Statistical Analysis

All statistical analyses were performed using R, version 3.5.3. (27) (RRID:SCR_001905).

## Results

All datasets used for this study are summarized in Table 1. Specifically, the comparison of the Swiss MS registry subgroup using DMT and the Swiss national MS treatment registry, Table 1 columns 2 and 3, as referred to in Step 3, shows some differences. The Swiss MS registry has a higher share of women (78.0 vs. 71.3%), fewer persons on first-line injectable treatments (24.5 vs. 31.4%) and a higher share of persons with a secondary-progressive MS (9.2 vs. 5.6%). The difference in the regional distribution disappeared after the extrapolation in Step 5, Table 1 column 4.

**Table 1:** Comparison of multiplier and benchmark datasets as well as external data.

			**Description**			
	**SMSR**	**SMSR**	**SVK**	**SVK augmented**	**Mediservice**	**SMSC**
	**All with CDMS**	**DMT in last 12 months**	**DMT in 2016**	**DMT in 2016**	**DMT in 2016**	**In care at MS centers**
*N*	1,567	973	6,057	9,503	4,613	1,115
Female	73.6%	78.0%	71.3%	71.2%	71.0%	65.8%
Median age (IQR)	48 (38–56)	44 (36–52)	45 (36–53)	45 (36–53)	–	41 (33–50)
Age Categories						
19–49	55.5%	68.2%	63.6%	65.6%	63.7%	–
50–64	34.5%	28.4%	30.5%	28.7%	30.2%	–
65 and older	10.1%	3.4%	5.9%	5.7%	6.1%	–
DMT status						
% never received DMT	13.8%	–	–	–	–	7.2%
% ever exposed to DMT	24.1%	–	–	–	–	12.6%
% receiving DMT in last 12 mt	62.1%	100%	100%	100%	100%	80.2%
DMT type						^*^
No treatment	37.9%	–	–	–	–	19.8%
Interferon/Glatiramer Acetate	15.2%	24.5%	31.4%	33.9%	38.8%	7.9%
Oral	37.1%	59.8%	56.8%	53.0%	57.1%	49.7%
Other infusion therapies	9.8%	15.7%	11.9%	13.0%	4.1%	21.1%
MS type						^**^
Relapsing-remitting MS	72.2%	90.8%	94.4%	94.8%	–	86.3%
Secondary-progressive MS	16.8%	9.2%	5.6%	5.2%	–	5.3%
Primary-progressive MS	10.9%	–	–	–	–	4%
Disease duration from FS (IQR)	11 (6–19)	9 (5–16)	8.0 (3.6–14.2)	8.1 (3.8–14.2)	–	10.0 (5.5–16.7)
Region						
Lake Geneva region	11.5%	11.2%	8.3%	14.1%	7.7%	–
Midlands	25.1%	24.9%	22.6%	24.6%	24.4%	–
North-western Switzerland	16.6%	16.0%	18.9%	16.5%	19.1%	–
Eastern Switzerland	14.1%	14.3%	17.1%	13.8%	17.7%	–
Ticino	3.5%	3.5%	0.9%	3.0%	5.5%	–
Central Switzerland	8.3%	8.4%	11.8%	9.5%	10%	–
Zurich	20.9%	21.7%	20.4%	18.5%	15.7%	–

*Columns 1 and 2 display the population characteristics of the Swiss Multiple Sclerosis Registry in total (column 1) and the subsample which used DMT in the last 12 months (column 2). Columns 3 and 4 display the information of the Swiss national MS treatment registry dataset as well as the augmented Swiss nation MS treatment registry dataset which represents the entire population of persons with MS using a DMT in 2016. Column 4 displays the characteristics of the Mediservice dataset, which is an aggregated dataset that contains information about persons with MS using the drug delivery and handling service of the MediService company. Column 5 shows aggregated data of the Swiss Multiple Sclerosis Cohort (SMSC) study, a clinic based, longitudinal cohort in Switzerland in 2016*.

* *1.5% have other DMTs*.

** *4.1% have a CIS. CDMS, Clinically Definite Multiple Sclerosis; DMT, Disease-Modifying therapies; FS, First Symptoms; IQR, Interquartile Range; MS, Multiple Sclerosis; SMSC, Swiss Multiple Sclerosis Cohort; SMSR, Swiss Multiple Sclerosis Registry; SVK, Swiss national MS treatment registry*.

The comparison of the Swiss national MS treatment registry with the MediService data, Table 1 columns 3 and 5, showed good agreement apart of fewer infusion therapies in the MediService data (11.9 vs. 4.1%) and some regional differences.

The Swiss MS registry compared to the Swiss MS cohort study, Table 1 columns 1 and 6, showed clear population differences, for example regarding median age (Swiss MS registry: 48, Swiss MS cohort study: 41), the treatment status (Swiss MS registry current: 62.1%, Swiss MS cohort study current: 80.2%) and the MS type (Swiss MS registry RRMS: 72.2%, Swiss MS cohort study RRMS: 86.3%).

For the estimation, all necessary parameters were available from either the augmented Swiss national MS treatment registry (${\hat{N}}_{x}=$ 9,503) or the Swiss MS registry ($\hat{\pi }$ = 62.1% and *n* = 1,567). Regarding the assumptions, the Swiss national MS treatment registry is not exhaustive, however, was extrapolated using MediService data to the augmented Swiss national MS treatment registry. The representativeness of the Swiss MS registry of the target population was considered by the dataset comparisons (Swiss national MS treatment registry and Swiss MS cohort study). The coinciding subgroups condition was fulfilled (step 3).

Because the two key parameters for the estimation (${\hat{N}}_{x}$ and $\hat{\pi }$) were not known precisely, they were sampled from a uniform distribution for the analysis to account for this uncertainty. The distribution width was defined to provide a conservative range on the basis of observed information and an additional margin reflecting the remaining uncertainty. The empirical estimates of the group size of the subgroup using DMT (${\hat{N}}_{x}$) mainly depended on the assumed Swiss national MS treatment registry coverage of Swiss PwMS and were derived from the MediService and the Swiss national MS treatment registry datasets. The observed Swiss national MS treatment registry coverage difference between the two databases was 2% (Swiss national MS treatment registry reported: 67%, MediService 65%). However, because the exact shares could not undoubtedly be identified, we decided for a conservative sampling distribution range of ${\hat{N}}_{x}$ +/- ${\hat{N}}_{x}*0.05$. For the percentage of persons with MS who used a DMT ($\hat{\pi }$), the confirmatory information consisted of Swiss MS registry data and previously reported information of a Swiss burden and cost of MS study by Calabrese et al. (28). The observed difference in the percentage of persons receiving DMT between the two databases was 1.4% (Swiss MS registry: 62.1%, Calabrese: 63.5%) (28). However, because the Calabrese study did not work with a prevalence sample and the disease course distribution was likely shifted, we decided to include a conservative range of $\hat{\pi }$ +/- $\hat{\pi }*0.05$.

**Figure 2:**
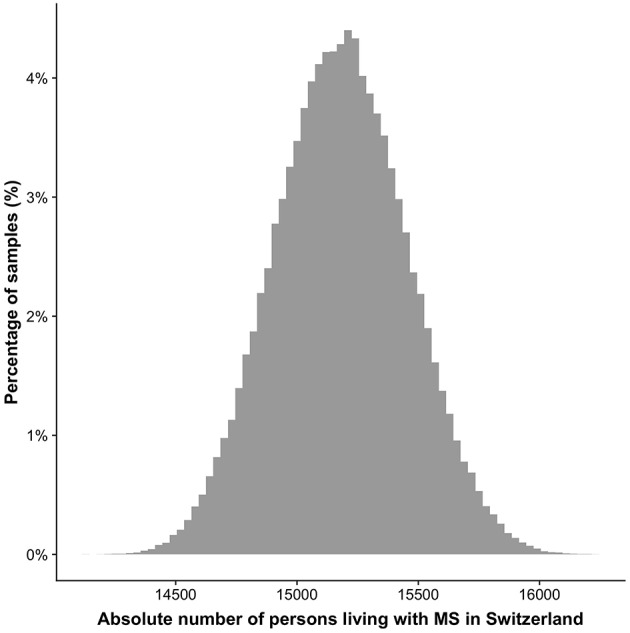
Distribution of the number of persons living with MS in Switzerland. The y-axis depicts the percentage of the 100,000 samples in a specific range based on the resampling approach. The 95% confidence interval reaches from 14,650 to 15,700.

The estimation resulted in a total number of 14,650–15,700 (95%-interval) PwMS living in Switzerland (period prevalence), Figure 2. The prevalence per 100,000 ranged from 174.0/100,000 to 186.7/100,000 (95%-interval).

**Figure 3:**
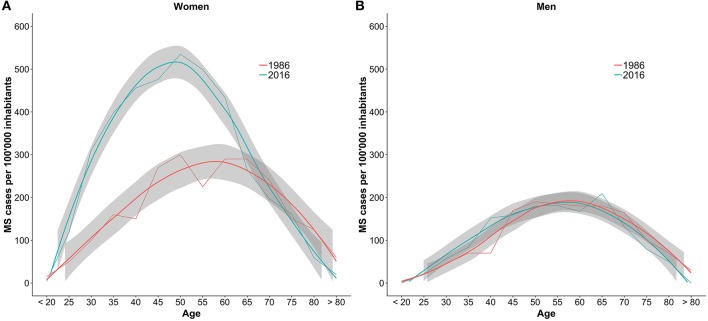
Comparison of the number of MS cases per 100,000 inhabitants per age group between 1986 and 2016. The numbers are stratified by sex with women in plot **(A)** and men in plot **(B)**. The gray bands are the result of smoother functions being applied to the data and do not represent confidence intervals of the estimates.

The comparison between the sex- and age-specific prevalence per 100,000 inhabitants in 1986 and 2016 is shown in Figure 3. In women until the age of 65 the prevalence increased by 150–300%.

The results were reviewed by an expert panel and approved as sensible with the slight exception of potential disease course shifts (mixture of secondary-progressive MS and primary-progressive MS).

## Discussion

This study proposes an estimation framework for the disease burden of chronic diseases and applied it successfully to estimate the prevalence of multiple sclerosis in Switzerland. A period prevalence between 14,650 and 15,700 (174.0/100,000–186.7/100,000) in 2016 was estimated and judged as reasonable by Swiss MS specialists. Compared to prior estimates from 1986, the number of MS diagnoses in women is higher, however, in men we did not detect a difference.

The newly obtained prevalence estimate of MS in Switzerland is substantially higher than the last estimate by Beer and Kesselring (4). The estimate is likely to be minimally larger still, because the SMSR only includes adult PwMS but not children. However, the expected number of persons with pediatric MS is low (29).

The prevalence per 100,000 fits well in with recent European estimates, which is reassuring as the Swiss population in 2016 has a similar age structure compared to the European standard population (20, 30). For example, the prevalence per 100,000 inhabitants in the UK (2010: 203.4/100,000), Sweden (2008: 188.9/,100,000) and Norway (2013: 208/100,000) were slightly higher (21–23). The estimates of Denmark (2005: 154.5/100,000) and France (2012: 151.2/100,000) were marginally smaller (1, 24). In contrast, the prevalence of the neighboring country Germany (Bavaria 2015: 277/100,000, Germany 2010: 289/100,000) was markedly higher (25, 26).

Furthermore, looking at the age- and sex-specific prevalences, the increase observed in this study coincides with higher number of diagnoses in women below the age of 65. This corresponds well to the finding of Koch-Henriksen et al. that the incidence of MS strongly increased in women, especially from 1980 on (31). This study, however, cannot disentangle the effect of increased incidence compared to improved diagnoses and increased awareness on the higher prevalence. The suggested increase of the incidence of late-onset MS cannot be seen from the Swiss data.

The potential causes of the observed increase in Switzerland are difficult to disentangle but could be related to the factors already discussed by Koch-Henriksen et al., namely a better and earlier case ascertainment in recent years, increased cigarette smoking, increased occurrence of obesity and fewer childbirths (31). However, the temporal trends of these risk factors only partially overlap with the observed age structure in this study, thus leaving room for the impact of other, currently unknown factors (32). In addition, the shift in demographics and increased life expectancy between 1986 and 2016 is likely to have increased the prevalence of MS.

Regarding the limitations, some short-comings on the data side need to be mentioned. The comparison of the Swiss MS registry subgroup with the Swiss national MS treatment registry dataset showed differences regarding type of DMT and the sex-ratio. Reasons for these differences might be the underlying insurance population covered and the potential underrepresentation of the Swiss MS registry in the tails of the age distribution. However, we are convinced that these factors have a minimal influence on the overall prevalence. The difference in the distribution of disease courses (relapsing-remitting MS vs. secondary-progressive MS) is likely to be due to misclassification in the Swiss national MS treatment registry database. Additionally, no single dataset is available for external verification of the Swiss MS registry distributions and as a consequence the estimation results. However, the observed differences between the Swiss MS registry to the Swiss MS cohort study validation dataset were expected and plausibly explainable. Moreover, there was general agreement regarding key variables in both databases and others studies, for example regarding the sex-ratio, age distribution, and MS type (21, 22, 29), which—overall—is reassuring. Lastly, the comparison between 1986 and 2016 is interesting but should be regarded critically. The studies were conducted using different study designs and target populations, in different times, which will likely impact the results. Especially in the tails of the age distributions, uncertainty is higher.

The estimation framework yielded a precise prevalence estimate for MS in Switzerland, but is not limited to the present use-case. The framework was constructed to be applicable to any chronic disease. The framework reduces the limitations of reliance on single datasets but also mitigates some caveats of estimation methods based on aggregation from multiple sources. Moreover, the integration of the critical appraisal step for results and methods ensures the integration of different expert perspectives which therefore lead to greater credibility of the estimation results, provided that critical expert appraisals are adequately reflected in the research report. The proposed Swiss estimation framework complements the recently introduced 4-step American framework by the United States Multiple Sclerosis Prevalence Workgroup for health care reimbursement claims (3). While our framework is generally generic and not specific to a certain type of data, options for independent case ascertainment—a key strength of the U.S. approach—are very limited. However, our framework easily can integrate information from algorithm-driven MS case identification in claims data as used by the U.S. study, thereby strengthening the evidence base.

We conclude that the number of persons diagnosed with MS in Switzerland increased considerably in the last 30 years and this increase could mainly be seen in women below the age of 65. Moreover, the use case demonstrates that the introduced estimation framework is well-applicable in the context of multiple sclerosis and helped to overcome common limitations of health insurance data or clinic-based estimations.

## Data Availability

The datasets generated for this study will not be made publicly available. The authors do not have the permission to share the datasets.

## Author Contributions

MK, MP, and VW contributed in the conception, the design of the study, performed the statistical analysis, the framework development, and wrote the first draft of the manuscript. All authors were involved in the data acquisition, interpretation and refinement of the results, the critical revision of the manuscript, and read and approved the submitted manuscript.

### Conflict of Interest Statement

JK received speaker fees, research support, travel support, and/or served on advisory boards by ECTRIMS, Swiss MS Society, Swiss National Research Foundation (320030_160221), University of Basel, Bayer, Biogen, Genzyme, Merck, Novartis, Protagen AG, Roche, Teva, Celgene. ÖY institution University Hospital Basel received grants from ECTRIMS/MAGNIMS, University of Basel, Pro Patient Stiftung University Hospital Basel, Free Academy Basel, Swiss Multiple Sclerosis Society and advisory board fees from Sanofi Genzyme, Biogen and Novartis Poland exclusively used for support of research and educational activities. PC has received honoraria for speaking at scientific meetings, serving at scientific advisory boards and consulting activities from Abbvie, Actelion, Almirall, Bayer-Schering, Biogen, EISAI, Lundbeck, Merck Serono, Novartis, Sanofi-Aventis, and Teva. He also receives research Grants from the Swiss Multiple Sclerosis Society (SMSG), and the Swiss National Research Foundation. TM is an employee of MediService. The remaining authors declare that the research was conducted in the absence of any commercial or financial relationships that could be construed as a potential conflict of interest.
