# Supplementary material for: A Framework for Estimating the Burden of Chronic Diseases: Design and Application in the Context of Multiple Sclerosis
Source: Front Neurol. 2019 Sep 4;10:953. doi: 10.3389/fneur.2019.00953 (PMC6742909; doi:10.3389/fneur.2019.00953)
Supplement: Supplementary file 1 [file Data_Sheet_1.pdf]

Supplementary Material

1 Augmented Swiss national MS treatment registry dataset:

The Swiss national MS treatment registry does not cover the entire population of PwMS using DMT in Switzerland but around 67% (year 2016). Instead of directly extrapolating the number by dividing it by 0.67, we decided to follow a more sophisticated approach by simulating the missing population using a resampling approach. The reasons for this were two-fold. First, the group size of previously underrepresented subgroups could be increased by using weights from an external dataset. Therefore, the credibility of the data could be improved. Second, the augmented dataset grants the possibility of later subgroup calculations while limiting the biases of an incomplete coverage.

Therefore, the percentage of persons served by the Swiss association for joint tasks of health insurances in specific subgroups of the MediService data was determined. The subgroups were specified by region of living (Lake Geneva Region, Midlands, North-western Switzerland, Eastern Switzerland, Ticino, Central Switzerland, Zurich), sex (female, male), age (18-49, 50-64, >65) and type of DMT (Injectables (first-line), Oral, Infusion therapies). In a next step, the same subgroups were defined in the Swiss national MS treatment registry dataset. Then the total size of these subgroups was calculated by dividing the group size of the subgroups in the Swiss national MS treatment registry by the Swiss association for joint tasks of health insurances share of these subgroups in the MediService data. Lastly the number of persons not yet covered were sampled with replacement from the existing ones.

To illustrate, we created an example: Let us assume the subgroup of persons living in the region of Zurich (region of living), are female (sex), are between 18 and 49 years old (age) and are taking an injectable first-line DMT (type of DMT) consists of 100 persons. The share of persons with Swiss

association for joint tasks of health insurances handling of the same subgroup in the MediService corresponds to 80%. Then the completed group consists of 125 persons (100 / 0.8). The 25 persons that are not in the Swiss national MS treatment registry dataset are consequently sampled with replacement from the 100. The augmented Swiss national MS treatment registry dataset is the result.

The approach has its limitations if certain persons are systematically not included in the Swiss national MS treatment registry. However, the increased credibility of the dataset in general and the possibility of subgroup calculations on a solid basis counterbalance these.
